# Optimized Design of a Hexagonal Equal Gap Silicon Drift Detector with Arbitrary Surface Electric Field Spiral

**DOI:** 10.3390/mi14101943

**Published:** 2023-10-18

**Authors:** Jiaxiong Sun, Zheng Li, Xiaodan Li, Manwen Liu, Hongfei Wang

**Affiliations:** 1 College of Physics and Optoelectronic Engineering, Ludong University, Yantai 264025, China; xiong98sjx@163.com (J.S.); m17852617552@163.com (X.L.); wanghf.20@163.com (H.W.); 2 School of Integrated Circuits, Ludong University, Yantai 264025, China; 3 Engineering Research Center of Photodetector Special Chip in Universities of Shandong, Ludong University, Yantai 264025, China; 4 School for Optoelectronic Engineering, Zaozhuang University, Zaozhuang 277160, China; 5 Institute of Microelectronics, Chinese Academy of Sciences (IMECAS), Beijing 100029, China; liumanwen@ime.ac.cn; 6 School of Physics and Physical Engineering, Qufu Normal University, Qufu 273165, China

**Keywords:** double-sided equal-gap spiral ring, X-ray detection, position resolution, electrical properties, carrier drift channel

## Abstract

In our previous studies, the silicon drift detector (SDD) structure with a constant spiral ring cathode gap (g) and a given surface electric field has been partially investigated based on the physical model that gives an analytical solution to the integrals in the calculations. Those results show that the detector has excellent electrical characteristics with a very homogeneous carrier drift electric field. In order to cope with the implementation of the theoretical approach with a complete set of technical parameters, this paper performs different theoretical algorithms for the technical implementation of the detector performance using the Taylor expansion method to construct a model for cases where the parameter “*j*” is a non-integer, approximating the solution with finite terms. To verify the accuracy of this situation, we performed a simulation of the relevant electrical properties using the Sentaurus TCAD tool 2018. The electrical properties of the single and double-sided detectors are first compared, and then the effects of different equal gaps g (g = 10 μm, 20 μm, and 25 μm, respectively) on the electrical properties of the double-sided detectors are analyzed and demonstrated. By analyzing and comparing the electrical characteristics data from the simulation results, we can show that the double-sided structure has a larger transverse drift electric field, which improves the spatial position resolution as well as the response speed. The effect of the gap size on the electrical characteristics of the detector is also analyzed by analyzing three different gap bifacial detectors, and the results show that a 10 μm equal gap is the optimal design. Such results can be used in applications requiring large-area SDD, such as the pulsar X-ray autonomous navigation. in the future to provide navigation and positioning space services for spacecraft deep-space exploration.

## 1. Introduction

The SDD, introduced in the 1980s [1], is based on the principle of lateral depletion, in which a bias voltage is applied to the front and back of the detector to completely deplete the lightly referenced substrate. After more than 40 years of development, it has become an important detector at the frontiers of physics, chemistry, and astronomy because of its high energy resolution, high precision, and high efficiency [2,3,4,5,6,7].

For example, large-area SDD can be used in the new space technology: X-ray pulsar navigation [8,9], a navigation system for spacecrafts travelling in deep space [10,11]. In previous studies, silicon microstrip detectors [12,13], concentric circle detectors [14], and silicon drift spiral bias adapters [15] have their own corresponding performance defects with excessive capacitance, inability to achieve automatic voltage division, high power consumption, heat generation, and short circuit. The spiral silicon drift detector calculates the optimal drift channel of carriers according to its physical model, and the resistance distribution of its spiral ring is related to the ion implantation concentration, the ring width, and the gap of the spiral ring itself [16]. One of the main tasks to achieve large-area SDD is to minimize its leakage current. In this paper, a new detector structure that controls the cathode gap of the spiral ring is used as the object of study. The cathode gap is kept small and constant to minimize the surface state and thus the surface leakage current, as a way to improve the detector performance. At the same time, we then reasonably adjust the given surface electric field while controlling the gap size constant to obtain an optimal carrier drift electric field in practical applications. This design has been partially investigated in our previous work [17], i.e., when “*j*” is an integer, the rotation angle *φ*(*r*) in Equation (13) in ref. [17] can be solved analytically by integrating each term. However, these analytical solutions are only a very small and limited part of all possible solutions. When “*j*” is an arbitrary number, we can obtain the complete set of solutions. We can therefore obtain the optimal detector structure from this set of solutions for different situations and achieve the best design of detection more comprehensively. In this paper, when *j* is not an integer, we approximate the solution of Equation (16) in ref. [17] with finite terms using the Taylor expansion in Equation (17) in ref. [17]. We use a computer-aided design (TCAD) tool to construct a model for the case where “*j*” is not an integer. To verify the accuracy of this case, we compare the electrical properties of the single- and double-sided detectors given an identical surface electric field in two cases (|*x*| > 1 and |*x*| < 1), and then, we compare the electrical properties of the double-sided detectors with detector with different equal gaps g on the electrical properties.

## 2. Detector Design and Modeling

**Figure 1:**
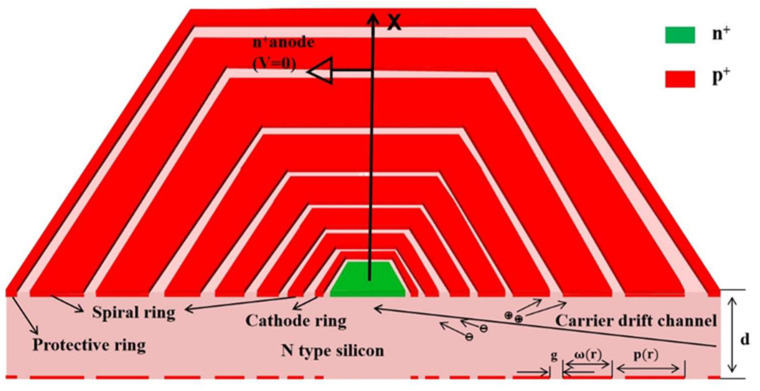
Schematic diagram of the detector structure.

The structure of the detector is shown in Figure 1 (due to the large size and complex structure of the device, the sizes of electrodes in the figure are not exactly the real sizes). In this design, we propose a hexagonal equal gap and arbitrary given surface electric field spiral silicon drift detector design. Ultra-pure high-resistivity silicon (UHS) with a thickness of 300 µm is used as a substrate with a resistivity greater than $4\times 10^{3}\;\Omega \cdot cm$ and an N-type silicon substrate (doping ~$4\times 10^{11}/cm^{3}$). There are two types of electrodes on the front of the detector (collection side): anode (n+) and cathode (p+). From the inside to the outside there are the anode, cathode ring, spiral ring, and protection ring, where the size of the anode is 60 µm with N-type heavy doping (doped phosphorus) concentration of $1\times 10^{19}/cm^{3}$ and a doping depth of 1 µm. The cathode ring has an inner diameter of 70 µm and an outer diameter of 90 µm (ring width of 20 µm). The spiral ring extends from the inside to the outside, and the ring width gradually increases with radius. The protection ring is formed at the outermost boundary, which can form a uniform electric field distribution at the edge of the detector, thereby avoiding the influence of inhomogeneous electric field in the edge region and protecting the performance of the detector edge. The cathodes are all P-type heavily doped (doped boron) with a doping concentration of $1\times 10^{19}/cm^{3}$ and a doping depth of 1 µm.

The incident side is the opposite side of the front side (the backside). The cathode on this side consists of a cathode ring, a spiral ring, and a protection ring, with the doping type and concentration the same as the those of the front side cathode, forming a double-sided symmetrical spiral structure. There are 0.1 µm SiO_2_ layer covering spiral rings on both front and backside silica layers on both sides. The electrode contacts are covered with a 1 µm aluminum layer on the anode, the cathode ring, the innermost starting positions and outermost ending positions of the spiral rings, and the protection ring [18]. After applying different bias voltages at the electrode contact points, a carrier drift channel is formed in the depletion region inside the device (N-type substrate), and a current signal is formed when the electrons generated by X-ray irradiation drift to the anode. This electrical signal is processed by a readout electronic chip (e.g., ATLAS detectors use ASICs to read signals from semiconductor and photonic detectors [19,20,21]) to produce an SDD charge signal.

To optimize the detector design for different cases of building structures, we can use Taylor expansions to approximate the finite term solution when “*j*” is not an integer, as mentioned in ref. [17]. According to Equation (13) of ref. [17], we know that the spiral angle *φ*(*r*) can be written as

(1) $$\varphi \left(r\right)=\frac{4\pi }{\beta g\sigma^{\frac{1}{\beta }}}\int_{y_{1}}^{y}\frac{(y-1{)}^{\frac{1-\beta }{\beta }}}{y}dy$$

where β is a variable, $y=1+\sigma r^{\beta }$, $y_{1}=1+\sigma r_{1}^{\beta }$, $r_{1}$ is the radius of the innermost ring of the spiral ring, r is the radius of the spiral ring, g is the ring spacing between two adjacent spiral rings, and σ is a quantity set for ease of derivation. If $j=\frac{1-\beta }{\beta }\neq \mathrm{integer}$*,* we have

(2) $$\varphi \left(r\right)=\frac{4\pi }{\beta g\sigma^{\frac{1}{\beta }}}\int_{y_{1}}^{y}\frac{y^{j}(1-\frac{1}{y}{)}^{j}}{y}dy$$

where $y=1+\sigma r^{\beta }$. If “*j*” is not an integer, since $\frac{1}{y}<1$,

(3) $$(1-\frac{1}{y}{)}^{j}=(1+z{)}^{j}$$

where $z=-\frac{1}{y}$. We define

(4) $$f(z)=(1+z{)}^{j}$$

The nth-order derivative is

(5) $$f{\left(z\right)}^{n}=j(j-1)\cdots (j-n+1)(1+z{)}^{j-n}\;\;\;\;(n=1,2,3\cdots )$$

Then, using Taylor expansion we have

(6) $$f\left(z\right)≅f\left(0\right)+\frac{f{\left(0\right)}^{\prime }}{1!}z+\frac{f{\left(0\right)}^{\prime \prime }}{2!}z^{2}+\cdots +\frac{f{\left(0\right)}^{n}}{n!}z^{n}+\cdots \;\;\;\;\;\;\;\;\;\;\;\;\;\;\;\;\;\;\;\;=1+z+\frac{j\left(j-1\right)}{2!}z^{2}+\cdots +\frac{j\left(j-1\right)\cdots \left(j-n+1\right)}{n!}z^{n}+\cdots (n=1,2,3\cdots )$$

Since $\left|z\right|<1$, this series should converge, and we obtain

(7) $$\varphi \left(r\right)=\frac{4\pi }{\beta g\sigma^{\frac{1}{\beta }}}\int_{y_{1}}^{y}\left[y^{j-1}-y^{j-2}+\frac{j\left(j-1\right)}{2!}y^{j-3}\cdots +{\left(-1\right)}^{n}\frac{j\left(j-1\right)\cdots \left(j-n+1\right)}{n!}y^{j-1-n}+\cdots \right]dy$$

Integrating Equation (7), we obtain

(8) $$\varphi \left(r\right)=\frac{4\pi }{\beta g\sigma^{\frac{1}{\beta }}}\left[\frac{1}{j}\left(y^{j}-y_{1}^{j}\right)-\frac{1}{j-1}\left(y^{j-1}-y_{1}^{j-1}\right)+\cdots +{\left(-1\right)}^{n}\frac{j\left(j-1\right)\cdots \left(j-n+1\right)}{n!\left(j-n\right)}\left(y^{j-n}-y_{1}^{j-n}\right)+\cdots \right]$$

Since y>1, as n→∞, it follows that $y^{j-n}\to 0$. Again from Ref [17], we can write the electric potential $\phi \left(r\right)$ as

(9) $$\phi \left(r\right)=\frac{\phi_{I}}{2\beta g^{2}\sigma^{\frac{2}{\beta }}}\int_{x_{1}}^{x}\frac{(x+1{)}^{n}}{x}dx+V_{E1}$$

Here, ${x=\sigma }^{2}r^{2\beta }-1$.$\;V_{E1}$ is the bias voltage at the starting position of the spiral ring,$\phi_{I}=4\rho_{s}\alpha I$,$\;\rho_{S}$ is the sheet resistance of the implantation layer of the spiral ring, and the value of α is determined by the spiral geometry [16]. For the hexagonal structure in this paper, *α* = 6, and I is the spiral current. There are two cases here, namely $\left|x\right|>1$ and $\left|x\right|<1$.

When $\left|x\right|>1$, we have

(10) $$\phi \left(r\right)=\frac{\phi_{I}}{2\beta g^{2}\sigma^{\frac{2}{\beta }}}\int_{x_{1}}^{x}{x^{n-1}(1+\frac{1}{x})}^{n}dx+V_{E1}$$

We can obtain

(11) $$\left(r\right)=\frac{\phi_{I}}{2\beta g^{2}\sigma^{\frac{2}{\beta }}}\left[\frac{1}{n}\left(x^{n}-x_{1}^{n}\right)+\frac{1}{n-1}\left(x^{n-1}-x_{1}^{n-1}\right)+\cdots +\frac{n\left(n-1\right)\cdots \left(n-i+1\right)}{i!\left(n-i\right)}\left(x^{n-i}-x_{1}^{n-i}\right)+\cdots \right]$$

Here, as i→∞, $x^{n-i},x_{1}^{n-i}\to 0$. From the surface potential ϕ(r) we obtain σ:

(12) $$\sigma =\left\{\frac{\phi_{I}}{2\beta g^{2}}\right.[\frac{1}{n}\left(x^{n}-x_{1}^{n}\right)+\frac{1}{n-1}\left(x^{n-1}-x_{1}^{n-1}\right)+\cdots +\frac{n\left(n-1\right)\cdots \left(n-i+1\right)}{i!\left(n-i\right)}\left(x^{n-i}-x_{1}^{n-i}\right)\;+\cdots ]{\left.\frac{1}{V_{out}-V_{E1}}\right\}}^{\frac{\beta }{2}}$$

When $\left|x\right|<1$, we have

(13) $$\phi \left(r\right)=\frac{\phi_{I}}{2\beta g^{2}\sigma^{\frac{2}{\beta }}}\int_{x_{1}}^{x}\frac{1}{x}\left[1+x+\frac{n(n-1)}{2!}x^{2}+\frac{n(n-1)(n-2)}{3!}x^{3}+\cdots +\frac{n\left(n-1\right)\cdots \left(n-i+1\right)}{i!}x^{i}+\cdots \right]$$

The points will give you

(14) $$\phi \left(r\right)=\frac{\phi_{I}}{2\beta g^{2}\sigma^{\frac{2}{\beta }}}\left[\ln\frac{x}{x_{1}}+x-x_{1}+\frac{n\left(n-1\right)}{2\times 3}\left(x^{3}-x_{1}^{3}\right)+\cdots +\frac{n\left(n-1\right)\cdots \left(n-i+1\right)}{i!\left(i+1\right)}\left(x^{i+1}-x_{1}^{i+1}\right)+\cdots \right]$$

Similarly, the surface potential ϕ(r) gives σ:

(15) $$\sigma =\left\{\frac{\phi_{I}}{2\beta g^{2}}\right.[\ln\frac{x}{x_{1}}+x-x_{1}+\frac{n\left(n-1\right)}{2\times 3}\left(x^{3}-x_{1}^{3}\right)+\cdots +\frac{n\left(n-1\right)\cdots \left(n-i+1\right)}{i!\left(i+1\right)}\left(x^{i+1}-x_{1}^{i+1}\right)\;+\cdots ]{\left.\frac{1}{V_{out}-V_{E1}}\right\}}^{\frac{\beta }{2}}$$

For the two cases $\left|x\right|>1$ and $\left|x\right|<1$, two corresponding sigmas are obtained, and they can be obtained according to Equation (8) as follows:

(16) $$r={\left\{\left.\left(\left.{\left(\frac{j\varphi (r)\beta g\sigma^{\frac{1}{\beta }}}{4\pi }+{\left(1+\sigma r_{1}^{\beta }\right)}^{j}\right)}^{\frac{1}{j}}-1\right)\right.\frac{1}{\sigma }\right\}\right.}^{\frac{1}{\beta }}$$

In this paper, we use the case of j=0.1 as an example for calculation. According to the above theoretical derivation process, first of all, we derive the convergent sigma by iteration according to the relationship between the ring gap and the ring width, the spiral pitch, and the known the given parameters. Using the Taylor expansion method, a convergent series is obtained, and the first term in Equation (8) is taken. We obtain a continuous function of r and φ(r), since it is an implicit function, and we apply the iterative method to obtain the relation between φ(r) and r. If we design a hexagonal spiral structure, then each π/3 will obtain an r vertex, and then connect the adjacent vertices to obtain the corresponding structure. Similarly, if we design a quadrilateral spiral structure, then every π/2 is connected by a corresponding r vertex. The design in this paper is a hexagonal structure, so every time we increase π/3 degrees, we will obtain a corresponding r vertex. Further, with the continuous increase in φ(r) and r, the spiral ring extends from the inside to the outside until the maximum radius is reached, and the structure of the spiral ring is generated. The structural design of the detector is derived from a theoretical model, and Figure 1 shows a complete demonstration of the detector structure, including doping, thickness, electrode, gap, etc.

## 3. Detector Electrical Characteristics Analysis

We simulate the electrical characteristics of the device using the Sentaurus device module in our simulation, where a negative bias voltage is applied to the front and back of the device corresponding to the device performance. For the double-sided spiral ring detector, −2 V is applied to the front cathode ring, −6 V is applied to the innermost position of the front spiral ring, −84 V is applied to the outermost position of the front spiral ring and the protection ring, −56 V is applied to the cathode ring at the opposite side center position and the innermost position of the opposite spiral ring, and −75 V is applied to the outermost position of the opposite spiral ring and the protection ring. As for the single-sided spiral ring structure, if the bias voltage is limited, we apply −2 V to the front cathode ring, −6 V to the innermost position of the front spiral ring, −35 V to the outermost position of the front spiral ring and the guard ring, and −33 V to the opposite structure with the whole side as the cathode. Using these two sets of bias voltages, we obtain the relevant electrical characteristics of the SDD. In the following, we analyze the electrical characteristics of the device in two parts, first by comparing the electrical characteristics of single- and double-sided detectors, and then by analyzing and demonstrating the effect of different equal gaps g on the electrical characteristics of double-sided detectors.

### 3.1. Performance Comparison between Single and Double-Sided Detectors

Before investigating the performance of the bifacial detector, a simulation of the performance of the single-sided detector was performed using the Sentaurus TCAD tool. The difference between the single-sided structure and the bifacial structure is that there is no spiral ring structure on the backside of the single-sided structure, and the entire side is a heavily doped P-type cathode with a doping concentration of $1\times 10^{19}/cm^{3}$ and a doping depth of 1 µm. The cathode is covered with a 1 µm thick aluminum layer to protect the electrode and act as the electrode contact. A comparative analysis of the electrical properties of the single and double-sided structures is presented below.

**Figure 2:**
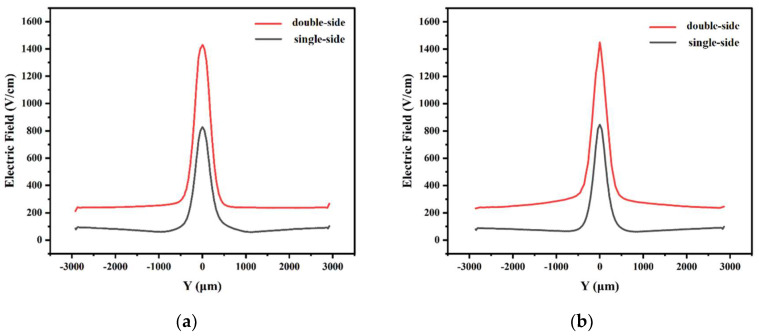
Comparison of electric fields in one-dimensional cross sections of double-sided and single-sided structures (at *z* = 160 μm): (**a**) $\left|x\right|>1$; (**b**) $\left|x\right|<1$.

Figure 2 shows the comparison of the one-dimensional electric field between the two-sided structure and the one-sided structure at the cut plane of *x* = 0 cross section, taken at *z* = 160 μm. For the two cases in Figure 2a,b, we can see from the plots that the electric field distributions of the two-sided structure and the single-sided structure are similar to a normal distribution, and the overall trend is to form a peak at the center anode, and the electric field gradually decreases from the highest at the center anode to both sides of the detector edge, showing a symmetrical distribution. However, the transverse drift electric field of the double-sided structure is larger than that of the single-sided structure, with a value of about 240 V/cm, while the electric field of the single-sided structure is about 100 V/cm. The double-sided structure has fewer low electric field regions, and the drift channel is more uniform and almost constant, which improves the carrier drift speed.

**Figure 3:**
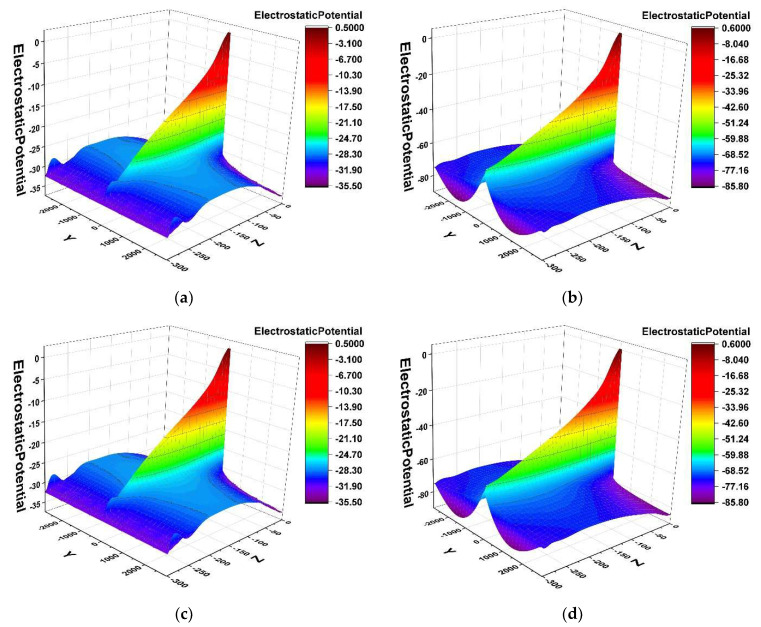
Comparison of two-dimensional potentials between double-sided and single-sided structures: (**a**) $\left|x\right|>1$ one-sided; (**b**) $\left|x\right|>1$ two-sided; (**c**) $\left|x\right|<1$ one-sided; (**d**) $\left|x\right|<1$ two-sided.

To see the gradient of the potential more intuitively, we plotted the two-dimensional potential surface distributions as shown in Figure 3 for the double-sided structure and the single-sided structure in the detector X = 0 (Y-Z positive plane) cross section of the detector. Figure 3a,c show the one-sided structure, and Figure 3b,d show the two-sided structure. From these four figures, we can clearly see that there are many equipotential surfaces on the surface, and one-by-one, the equipotential surfaces are stacked to form a “ridge” style. In the simulation, we apply a negative bias voltage, from top to bottom, the potential gradually decreases, forming a potential gradient on both sides of the anode symmetrically, which is equivalent to the formation of a potential network on the surface. The electrons are transported to the anode on this potential network and finally collected at the anode. From the surface, we can see that the double-sided structure surface is smoother, showing a more uniform potential distribution. Double-sided structure isotropic lines are more compact. Double-sided potential is about three times the potential single-sided potential, meaning it can provide high potential. A double-sided spiral can therefore improve the collection time of electrons (therefore high drift field) compared to a single-sided structure.

**Figure 4:**
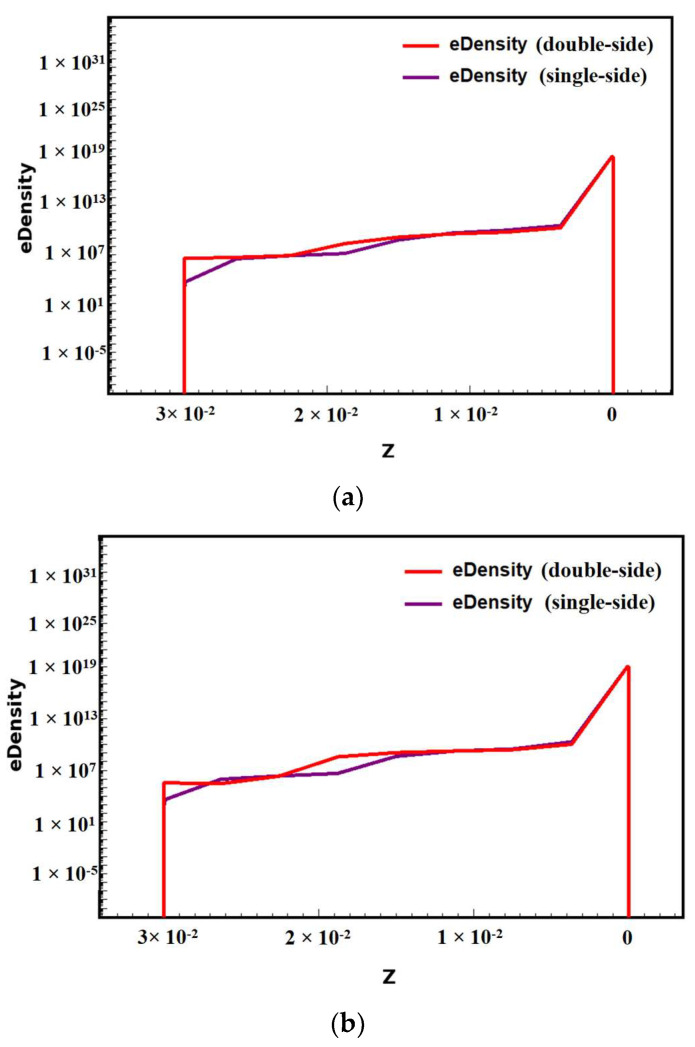
Comparison of electron concentrations in one-dimensional cross sections of double-sided and single-sided structures (at y = 0 μm): (**a**) $\left|x\right|>1$; (**b**) $\left|x\right|<1$.

Through the simulation results, we analyze the one-dimensional electron concentration comparison plots of the double-sided structure and the single-sided structure at *x* = 0 cross section, taking *y* = 0 μm. From Figure 4, we can see that the electron concentration gradually increases from the back side to the front side of the detector to the anode, and although the electron concentration is relatively large, it is still lower than the original doping concentration, indicating that the detector is completely depleted. Combining Figure 2 and Figure 3, we know that the incident particles first move into the drift channel driven by the high electric field of the double-sided structure, and then drift to the central collector anode driven by the nearly constant electric field in the drift channel. From Figure 4, we can see that the curve of the double-sided structure is more stable and smoother than the curve of the single-sided structure, so the double-sided structure improves the response speed [2].

By analyzing the electrical characteristics of the simulation results of the single-sided structure and double-sided structure, comparing with electric field, potential, and electron concentration, respectively, we conclude that the transverse drift electric field of double-sided structure is larger, the working voltage is larger, the low electric field region is less, and the drift channel is more uniform than those of single-side structure. Results of these comparison studies show that double-sided structure detector performances are better than single-sided ones, so in the next section, we analyze the double-sided structure as the research object.

### 3.2. Effect of Cathode Gap Size on the Electrical Characteristics of Double-Sided Detectors

Initially, double-sided SDDs were widely used as position sensitive detectors in particle physics [1,22,23]. In this paper, a novel detector structure for controlling the spiral ring cathode gap is investigated, i.e., keeping the gap small and constant to minimize the surface state and thus the surface leakage current, as a way to improve the detector performance. Three different double-sided detectors with equal gaps of 10 μm, 20 μm, and 25 μm, respectively, are selected to analyze the effect of the gap size on the electrical properties of the detector.

**Figure 5:**
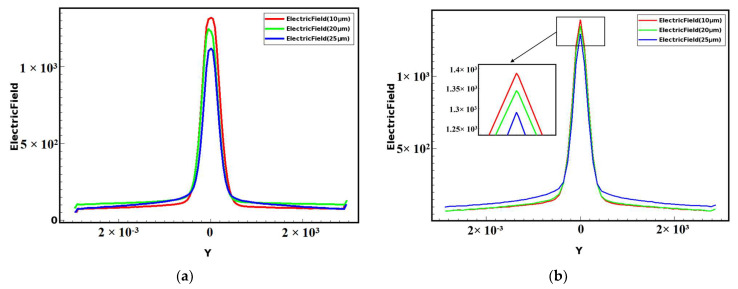
Comparison curves of electric fields of different gaps of double-sided detectors (at *z* = 190 μm): (**a**) $\left|x\right|>1$; (**b**) $\left|x\right|<1$.

First of all, we compare the electric field. From Figure 5, we can clearly see that at *z* = 190 μm, the smaller the gap, the higher the electric field value can reach at the center anode position. The highest electric field at the anode position decreases with the increase in the gap, and the highest electric field value decreases faster. On the other hand, the electric field curve is flatter and more uniform at the double-sided spiral ring position, as the gap decreases, giving a more uniform drift field.

**Figure 6:**
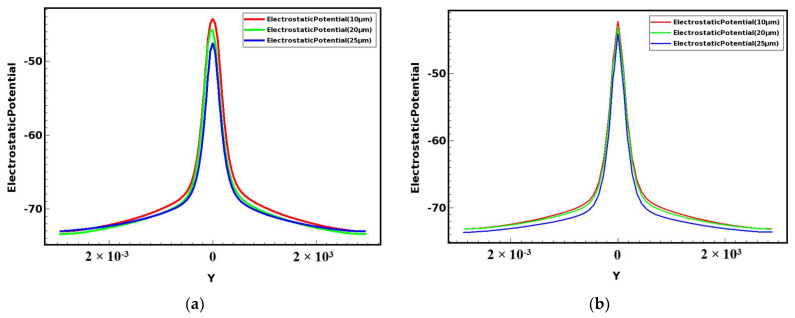
Comparison curves of different gap potentials of double-sided detectors (at *z* = 160 μm): (**a**) $\left|x\right|>1$; (**b**) $\left|x\right|<1$.

The gradient of the potential is the electric field; Figure 5 and Figure 6 are curve shape. Checking the correctness of the potential and electric field distribution, from the Figure 6 curve, we can see that the smaller the gap, the higher the value of its potential, and for the curve along the middle anode position to the edge, the potential falls more slowly. In the two-dimensional potential diagram such as Figure 3b,d, we can see that the smaller the gap, the more compact the distribution of the potential gradient. Then, the smaller the gap, the more uniform the potential gradient is, and the faster the incident particles or light reach the anode in the carrier drift channel to be collected.

**Figure 7:**
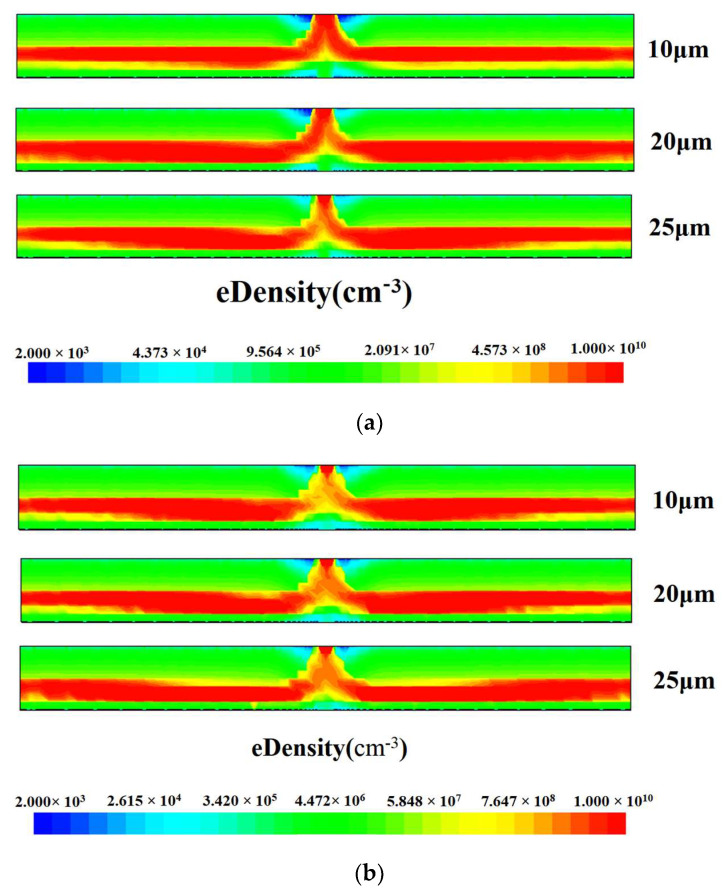
Comparison of electron concentration within different gaps of the double-sided detector: (**a**) $\left|x\right|>1$; (**b**) $\left|x\right|<1$.

By simulating the detector with different gaps, we obtained the X = 0 cross-sectional electron concentration comparison diagram shown in Figure 7, from which we can see that the electron concentration distribution inside the detector is almost symmetrical, and the electron concentration in the center of the detector is much higher than that on both sides. The electron drift path is clearly visible in the electron concentration diagram (red area in the diagram), which is also called the electron drift channel. But in the diagram, we can see the difference between the electron drift channels of different gaps. The smaller the gap, the narrower and flatter the electron drift channel, so the smaller the gap, the better the electron drifting towards the anode.

Therefore, combined with the above electrical property data, in general, equal cathode ring gap detectors show better electrical properties of SDD with smaller gaps, so we should design to reduce the gap g as much as possible. However, in the real SDD fabrication, the gap size is limited by the process technology, and if the gap is too small, the field between two adjacent rings may be large enough to cause surface breakdown. Therefore, we choose a gap of 10 μm as the minimum gap parameter based on theoretical calculations and the feasibility of our process while avoiding the risk of failure.

## 4. Conclusions

In this paper, we develop a simulation model of a hexagonal equal-gap and arbitrarily given surface electric field spiral-type silicon drift detector and its optimal design. That is, by keeping the gap small and constant, we improve the detector performance by minimizing the surface state and hence the surface leakage current for a given surface electric field. We use the computational design tool Sentaurus TCAD to approximate the model with finite term solutions using Taylor expansions for the case where “*j*” is not an integer. The electrical properties of the detector, including the distribution of electric field, potential, and electron concentration, are obtained, and electrical properties of the single and double-sided detectors in the cases of $\left|x\right|>1$ and $\left|x\right|<1$ have been compared. A comparison study on electrical characteristics between the double-sided structure and single-sided structure has shown that the double-sided structure detector performance is better than the single-sided performance. Meanwhile, we select three different gap double-sided detectors with equal gaps of 10 μm, 20 μm, and 25 μm to analyze the influence of gap size on the electrical characteristics of the detector, and the results show that the equal gap of 10 μm is the optimal design.

## Data Availability

Not applicable.
